# Meat hygiene knowledge, handling practices and associated factors among meat handlers in Gedeo zone, Ethiopia

**DOI:** 10.1038/s41598-023-42225-8

**Published:** 2023-09-13

**Authors:** Zemachu Ashuro, Nathnael Zeysse, Mulugeta Ayalew

**Affiliations:** 1 School of Public Health, College of Health Science and Medicine, Dilla University, Dilla, Ethiopia; 2 Department of Preventive Medicine, School of Public Health, College of Health Sciences, Addis Ababa University, Eastern Africa GEO Health Hub for Research and Training Project, P.O. Box 9086, Addis Ababa, Ethiopia

**Keywords:** Health care, Risk factors

## Abstract

A cross-sectional study was conducted among 239 randomly selected meat handlers working in butcher shop in southern Ethiopia to assess factors associated with meat hygiene knowledge and practices. A binary logistic regression analysis with a 95% confidence interval (CI) and a p-value < 0.05 was used to identify factors that were significantly associated with good level of meat hygiene knowledge and practices. The findings revealed that 38.5% [95% CI: 32.2–44.8%] and 25.1% [95% CI: 19.7–30.5%] of meat handlers have good levels of meat hygiene knowledge and practices, respectively. Good level of meat hygiene knowledge was significantly (p < 0.05) associated with educational level, having meat hygiene training, and having regular supportive supervision by health workers, whereas good level of meat handling practice was significantly associated with work experience, educational level, have regular supportive supervision by health professionals, and having meat hygiene training. In conclusion, the majority of meat handlers have poor knowledge and practices regarding meat hygiene among meat handlers. Educational level, meat hygiene training, and supportive supervision by a health professionals were all independent predictors of meat hygiene knowledge and practice among meat handlers. As a result, health professionals should give regular training, butcher shop inspections, and supportive supervision for meat handlers in order to improve meat hygiene knowledge and practices among meat handlers.

## Introduction

Access to safe and nutritious food is critical for sustaining life, preventing disease, and promoting good health. Unsafe food containing pathogens such as bacteria, viruses, parasites, or chemical substances can cause over 200 different diseases ranging from diarrhoea to cancer^1,2^. Foodborne disease (also known as foodborne illness or food poisoning) refers to any illness caused by consuming or ingesting contaminated food containing pathogenic bacteria, viruses, or parasites^3^. Every year, an estimated 600 million people, or nearly one in ten, become ill after eating contaminated food, resulting in 420,000 deaths and the loss of 33 million healthy life years (DALYs)^1,2,4^.

Meat is the most perishable food and an ideal medium for the growth and multiplication of microorganisms, which results in meat spoilage and, ultimately, food borne illness^5,6^. The main sources of pathogens or disease-causing organisms in meat and meat products are unhealthy animals, the personal hygiene and health of meat handlers, keeping food at temperatures favorable for microorganism growth, unsanitary food preparation and serving utensils, unsafe meat transportation, storage, and processing, unsanitary working conditions, and the use of polluted water for food preparation^6–10^.

Meat hygiene refers to a set of activities that need the implementation of specific standards, codes of practice, and regulatory action by the regulatory body to ensure the safety of the meat for consumers to eat. Hygiene requirements must be met at different stages of production, processing, and transportation, as well as for meat handlers, slaughter and meat processing equipment, and the environment^11,12^. In developing countries, poor food handling practices (for example, storing cooked and uncooked meat together allows microorganisms to travel from raw meat to cooked meat, resulting in cross contamination), an unsanitary work environment, a lack of medical checkups for meat handlers, poor personal hygiene of food handlers, and a lack of safe water for food preparation are major contributors to food borne diseases^11,13–15^. Food safety is a critical public health issue in all countries. Meat handlers' food safety knowledge, attitudes, and practices are a major source of concern. Different studies found that the magnitude of meat handlers with good food safety knowledge was 20% in Bangladesh^16^, 61.7% in Malaysia^9^, and 71.1% in Lagos State, Nigeria^17^, while the magnitude of meat handlers with good food safety practices was 25.5% in north-central Nigeria^18^, 16.3% in Bangladesh^16^, 66.6% in Lagos State, Nigeria^17^, and 77.7% in Malaysia^9^. However, in Ethiopia, the magnitude of meat handlers with good food safety knowledge ranges from 11.1% in Amhara National Regional State^19^ to 72.4% in Bishoftu City, Ethiopia^20^, while food safety practices range from 1.1% in Jigjiga Town, Ethiopia^21^ to 66.4% in Gondar Town^14^. In Ethiopia, the need for meat products is increasing rapidly and consuming raw meat has become a status symbol^22^. There are around 300 small slaughterhouses in Ethiopia that supply meat for local consumption, each with a different capacity and facility, but all with inadequate essential hygiene and sanitation facilities^23^. In Ethiopia, very few studies on food safety knowledge and practices of meat handlers have been conducted, but none of them have focused on assessing the factors associated with food safety knowledge and practices of meat handlers. Therefore, it is critical to assess meat handlers' level of meat hygiene knowledge, practices, and associated factors in order to identify risk factors and implement meat hygiene standards, codes of practice, and guidelines to prevent the spread of meat borne disease.

## Materials and methods

### Study design, setting and period

A cross-sectional survey with structured observation was conducted among butcher shop meat handlers in Dila town, Gedeo zone, southern Ethiopia, from June 5 to July 15, 2022. Dilla town is the capital of Gedeo zone and is located 365 km south of Ethiopia's capital city, Addis Ababa. The total population of Dilla is estimated to be 102,624, of these 50,286 are males and 52,338 are females. Livestock production is an essential agricultural sector in Ethiopia, accounting for around 18% of total GDP^24,25^. According to the Central Statistical Agency (CSA) (2021), the Gedeo zone has an estimated livestock population of 133,925 cattle, 197,846 sheep, and 22,621 goats^26^.

### Sample size determination

The sample size was determined using single and double population proportion formulae for three objectives (meat hygiene knowledge, practices, and associated factors among meat handlers). The single population proportion formula yielded the highest sample size for objective one (knowledge about meat hygiene), using the following assumptions: a 95% confidence interval (CI) and a 5% margin of error (d), and by taking a proportion of good level of meat handling knowledge 52.2% from previous study conducted in North Shewa Zone, Oromia, Ethiopia^27^.

$$\mathrm{n }= \frac{{\mathrm{Z\alpha }}^{2}\mathrm{p}(1-\mathrm{p})}{{\mathrm{d}}^{2}} = \frac{{1.96}^{2}\times 0.522(1-0.522)}{{0.05}^{2}} = 377.$$

We used the following correction formula to compute the adjusted sample size (because the total number of meat handlers working at butcher shops in the selected areas was < 10,000), and after adding a 10% non-response rate, the final sample size for this study was 242.

$${\mathrm{N}}_{\mathrm{f}} = \frac{Ni}{1+Ni/N}= 377/1+377/528 = 220 .$$

where Ni is the initial sample size, N_f_ is the final sample population.

### Sampling procedure

The list of existing butcher shops in Gedeo zone was obtained from the Gedeo Zonal Health Department's Health, Health Related Product Quality Control Authority. According to Health Related Product Quality Control Authority data, there were 179 butcher shops in Gedeo Zone in 2022. In this study, we randomly selected 100 butcher shops from a total of 179 butcher shops. Study participants were proportionately allocated to each selected butcher shops based on the number of meat handlers. Then, the sampling frame was prepared for each selected butcher shops using the updated list of meat handlers. Finally, study participants were selected using a simple random sampling technique from each establishment.

### Data collection tools and procedures

Data was collected using a semi-structured, pretested questionnaire. The questionnaires include socio-demographic information, meat handlers' meat hygiene practices and knowledge, as well as factors associated with meat handling knowledge and practices. The questionnaire was adapted from previously published research works^13,21,28,29^, translated to Amharic for ease of understanding during data collection, and then retranslated back to English to ensure consistency. The questionnaires were administered face to face by a trained health professional.

### Measurement of variables

The primary outcome variables were meat hygiene knowledge and practices among meat handlers. We used 14 closed-ended questions with three alternative answers (such as "True," "False," and "Don't know") to assess meat hygiene knowledge and practices among meat handlers. The minimum and maximum score for meat hygiene knowledge and practices were 0 and 14, respectively.

Level of meat hygiene knowledge: If the meat handler scored less than 70% on meat hygiene knowledge related questions (answered less than 10 questions out of 14 questions), the meat handler was considered to have a "poor level of meat hygiene knowledge." If a meat handler scored 70% or higher on the meat hygiene knowledge questions (answered 10 questions or more out of 14 questions), the meat handler was considered to have a "good level of meat hygiene knowledge"^14,21,30^.

Level of meat hygiene practices: If the meat handler scored less than the 70% of meat hygiene practice related questions (answered below 10 questions out of 14 questions), the meat handler considered as having a “poor level of meat hygiene practices.” If a meat handler scored 70% or higher on the meat hygiene practice-related questions (answered 10 or more questions out of 14), the meat handler was considered to have a "good level of meat hygiene practices”^14,21,30^.

### Statistical analyses

Data were analyzed using Stata version 17.0 (Corporation, College Station, TX: StataCorp LLC) and presented using frequencies and percentages. A multivariable binary logistic regression model was used to identify factors associated with meat handlers' food safety knowledge and practice. Adjusted odds ratios (AOR) with 95% confidence intervals (CI) and p-values < 0.05 were used to declare statistically significant associations. Independent variables with p < 0.25 in the univariate analysis were included in the final multivariable logistic regression model. The variance inflation factor (VIF) was used to test multicollinearity; the result was less than 2, and the model fitness was checked using the Hosmer and Lemeshow goodness-of-fit test; the result was 0.72.

### Ethical approval and consent to participate

The study was conducted after receiving ethical clearance from the Institutional Review Board (IRB) of the College of Health Science and Medicine, Dilla University (reference number: 017/2019; and protocol unique number: 002/19-11). We obtained written informed consent from meat handlers. All methods were performed in accordance with the relevant guidelines and regulations.

## Results

### Socio-demographic characteristics of study participants

This study included all 239 randomly selected study participants, with a 98.8% response rate. The majority of the study participants 149 (62.3%) were male with the mean (± SD) age of 27 (± 5.16) years. The majority of the study participants, 163 (68.2%), had completed primary education, and more than half (52.3%) were married, with a mean monthly income of 2923.01 Ethiopian birr. More than half of the study participants (56.1%) had worked for less than 5 years, and the majority of study participants 214 (89.5%) worked more than 8 h per day. However, 91 (38.1%) of participants had received meat hygiene training, and 84 (35.1%) of participants had regular medical checkups (Table 1).

**Table 1:** Socio-demographic characteristics of study participants in Gedeo Zone, Southern Ethiopia, 2022 (n = 239).

Variables	Category	Frequency (n)	Percentage (%)
Gender	Male	149	62.3
	Female	90	37.7
Age	≤ 27 years	154	64.4
	> 27 years	85	35.6
Marital status	Not married	114	47.7
	Married	125	52.3
Education status	Primary	163	68.2
	Secondary and above	76	31.8
Monthly income	≤ 2000 ETB	106	44.4
	> 2000 ETB	133	55.6
Working hour per day	≤ 8 hours	25	10.5
	> 8 hours	214	89.5
Meat hygiene training	Yes	91	38.1
	No	148	61.9
Regular medical check-up	Yes	84	35.1
	No	155	64.9
Supervision by health professional	Yes	103	43.1
	No	136	56.9
Work experience	< 5 years	120	50.2
	5–10 years	96	40.2
	> 10 years	23	9.6
Job description	Butcher	87	36.4
	Helper	67	28.0
	Cooker	85	35.6

*ETB:* Ethiopian Birr.

### Meat hygiene knowledge among meat handlers

In this study, almost all of the participants, 228 (95.4%), knew that washing hands reduces the risk of meat contamination. The majority of participants, 195 (81.6%) knew that insect such as cockroach and flies can contaminate row meat, and 207(86.6%) of study participants knew that food contamination is caused by unclean instruments and work surfaces. However, nearly half of the participants (48.1%) are unaware that healthy meat handlers can be carriers of pathogens found in food, 133 (55.6%) study participants didn’t know contaminated meat can cause meat borne disease, 97 (40.5%) are unaware that meat handlers with open wounds, gastroenteritis, or diseases of the ear or throat should not handle meat, and three fourth (75.7%) of the study participants didn’t knew the ideal temperature for storing fresh meat (Table 2).

**Table 2:** Meat hygiene knowledge of meat handlers in Gedeo zone, southern Ethiopia, 2022 (n = 239).

Variables	Response	Frequency (n)	Percent (%)
Consumer health risks could result from improper handling of meat	Yes	230	96.2
	No	5	2.1
	Don’t know	4	1.7
Hand washing before and during meat handling reduces the risk of food contamination	Yes	228	95.4
	No	3	1.3
	Don’t know	8	3.3
Using gloves when handling meat reduces the risk of meat contamination	Yes	98	41.0
	No	67	28.0
	Don’t know	74	31.0
Meat contamination risk is decreased by thoroughly cleaning and sanitizing knives and hooks	Yes	70	29.3
	No	47	19.7
	Don’t know	122	51.0
When workers eat and drink in the workplace, the risk of meat contamination increases	Yes	44	18.4
	No	183	76.5
	Don’t know	12	5.1
Food contamination is caused by unclean instruments and work surfaces	Yes	207	86.6
	No	3	1.2
	Don’t know	29	12.2
Even healthy food handlers can be carriers of pathogens found in food	Yes	27	11.3
	No	97	40.6
	Don’t know	115	48.1
Cockroaches and flies can contaminate raw meat	Yes	195	81.6
	No	16	6.7
	Don’t know	28	11.7
Employees of food and drinks establishments should have regular medical check-up	Yes	107	44.8
	No	47	19.7
	Don’t know	85	35.5
Meat-borne diseases such as *Shigellosis*, *Salmonella, E. coli, Diarrhea, Anthrax, and Brucellosis* can be caused by contaminated meat	Yes	100	41.8
	No	6	2.5
	Don’t know	133	55.6
Food handlers with open wounds, gastroenteritis, or diseases of the ear or throat should not handle meat	Yes	74	30.9
	No	68	28.4
	Don’t know	97	40.5
When microorganisms from contaminated meat are transferred to another by the food handler’s hand or utensils, cross contamination occurs	Yes	111	46.4
	No	27	11.3
	Don’t know	101	42.3
The ideal temperature for storing fresh meat is between 28° F and 32° F	Yes	48	20.1
	No	10	4.2
	Don’t know	181	75.7
A change in color, odor, or taste is always present in contaminated meat	Yes	140	58.6
	No	40	16.7
	Don’t know	59	24.7
When handling meat, follow food safety guidelines	Yes	88	36.8
	No	30	12.6
	Don’t know	121	50.6

### Attitude of meat handlers towards meat hygiene

The majority of the study participants reported that positive attitudes toward storing raw and cooked food separately (89.1%), regular meat safety training (83.4%), covering nose or mouth, during sneezing and coughing (61.4%), food handlers with abrasions or cuts on their hands should not handle food without gloves (69.8%), regular medical examination for the meat handlers (95.8%), wearing personal protective equipment (71.1%), disinfection and clean working surfaces and utensils with safe water (86.7%), and washing hands with soap and water (90.7%). However, 50.2%, 55.2%, and 45.9% of the study participants disagreed that raw meat and raw vegetables should never be cut with the same knife or cutting board, that wearing jewelry like watches, earrings, and rings increases the risk of meat contamination, and that the temperatures of refrigerators and freezers should be checked frequently, respectively (Table 3).

**Table 3:** Attitude towards meat hygiene practices among meat handlers in Gedeo Zone, Southern Ethiopia, 2022 (n = 239).

Variables	Response	Frequency (n)	Percent (%)
To reduce the risk of food contamination, raw and cooked foods should be stored separately	Agree	213	89.1
	Disagree	15	6.3
	Not sure	11	4.6
Raw vegetables and raw meat should never be cut on the same cutting board or with the same knife	Agree	93	38.9
	Disagree	120	50.2
	Not sure	26	10.9
Without covering our noses or mouths, sneezing and coughing could contaminate the meat	Agree	147	61.4
	Disagree	79	33.1
	Not sure	13	5.5
Food handlers with abrasions or cuts on their hands should not handle food without gloves	Agree	167	69.8
	Disagree	55	23.1
	Not sure	17	7.1
A medical examination every six months is required for the health of meat handlers	Agree	229	95.8
	Disagree	6	2.5
	Not sure	4	1.7
The chance of meat contamination rises when jewellery such as watches, earrings, and rings are worn	Agree	99	41.4
	Disagree	132	55.2
	Not sure	8	3.4
Personal protective equipment (clothing, shoes, and a hair cover) could improve workplace safety and hygiene practices	Agree	170	71.1
	Disagree	61	25.5
	Not sure	8	3.4
After disinfection, it is critical to clean working surfaces, cutting tools, knives, and hooks with safe water	Agree	207	86.7
	Disagree	32	13.3
	Not sure	0	0
Washing hands with soap can reduce meat contamination or food poisoning	Agree	217	90.7
	Disagree	22	9.3
	Not sure	0	0
Regular training and awareness programs improve meat safety handling practices while lowering the risk of contamination	Agree	199	83.4
	Disagree	22	9.2
	Not sure	18	7.4
Refrigerators and freezers should have their temperatures checked on a regular basis	Agree	98	41
	Disagree	110	45.9
	Not sure	31	13.1

### Meat hygiene practice of meat handlers

The vast majority of study participants practice unsanitary meat handling. More than half of the study participants, 71.5%, 68.2%, and 63.6%, respectively, did not use gloves during work, did not wash their aprons after each day’s work, and did not wear a mask during work. In addition, 53.1% of meat handlers did not wear a cap while working, 60.3% use and paint nail polish while handling meat, and 59.8% handled money while processing meat. Furthermore, 51.5% of participants reported that they handled meat when they had an illness and 79.9% of participants handled meat when they had cuts, wounds, bruises, or injuries on your hands. However, the majority of study participants (83.3%) reported washing their hands with water and soap before and after touching raw meat, 95.0% after using the toilet, and 58.2% after smoking, sneezing, or coughing (Table 4).

**Table 4:** Meat hygiene practice of meat handlers in Gedeo zone, southern Ethiopia, 2022 (n = 239).

Questions statement	Good practice		Poor practice	
	n	%	n	%
Do you wear gloves while handling meat?	68	28.5	171	71.5
Do you handle money while processing meat?	96	41.2	143	59.8
Do you wash your hands before and after handling or touching raw meat?	199	83.3	40	16.7
Do you wash your hand with water and soap after using the toilet?	227	95.0	12	5.0
Do you wash your hand after smoking, sneezing, or coughing?	139	58.2	100	41.8
Do you wear an apron while working?	203	84.9	36	15.1
Do you clean your aprons after each day of work?	76	31.8	163	68.2
Do you wear a mask while working?	87	36.4	152	63.6
Do you wear a cap while working?	112	46.9	127	53.1
Do you use and paint nail polish when handling meat?	95	39.7	144	60.3
Do you remove your work equipment when using the toilets?	160	66.9	79	33.1
Do you remove your personal items such as rings, necklaces, watches, and so on while working with meat?	105	44.1	134	56.1
When you’re sick, do you process or handle meat?	116	48.5	123	51.5
Do you handle or process meat if your hands are cut, bruised, or injured?	48	20.1	191	79.9

**Figure 1:**
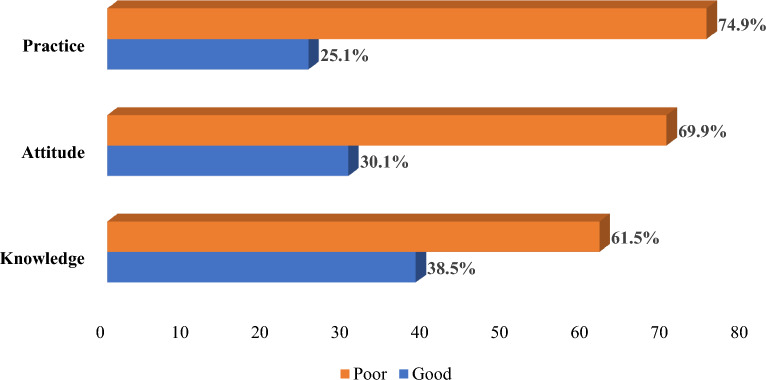
Meat hygiene knowledge, attitudes, and practices of meat handlers.

In this study, the overall mean and standard deviation for food safety attitudes among meat handlers was 0.31 ± 0.462, indicating that 30.1% of meat handlers had positive attitudes toward food safety practices, the overall mean and standard deviation (SD) for meat safety knowledge was 0.62 ± 0.488, indicating that 38.5% of meat handlers had good knowledge toward hygienic meat handling practices, and the overall mean and standard deviation (SD) for hygienic meat handling knowledge was 0.75 ± 0.435, indicating that 25.1% of meat handlers had good knowledge towards meat hygiene practices among meat handlers (Fig. 1).

### Factors that associated with meat hygiene knowledge among meat handlers

Variables with p-values less than 0.25 in bivariable logistic regression analysis, such as gender, age, educational level, job type, work experiences, supervision by health workers, and meat hygiene training, were candidates for multivariable logistic regression analysis. Gender, educational status, supervision by health workers, and meat hygiene training were significantly associated with meat hygiene knowledge among meat handlers with p-values less than 0.05 in multivariable logistic regression analysis.

Workers with a secondary education or higher were 2.26 times more likely to have a good level of meat hygiene knowledge than their counterparts [AOR 2.26, 95% CI: 1.18–4.33, p-value = 0.014], workers who had meat hygiene training were 1.97 times more likely to have a good level of meat hygiene knowledge than workers who did not have meat hygiene training [AOR 1.97, 95% CI: 1.04–3.75, p-value = 0.038], workers who were supervised by a health professional were 3.05 times more likely to have a good level of meat hygiene knowledge than those who were not [AOR 3.05, 95% CI: 1.53–6.07, p-value = 0.002]. However, male workers were 0.38 times less likely have a good level of meat hygiene knowledge than females [AOR 0.38, 95% CI: 0.21–0.71, p-value = 0.002] (Table 5).

**Table 5:** Factors associated with meat hygiene knowledge among meat handlers in Gedeo zone, Southern Ethiopia, 2022 (n = 239).

Variables	Meat hygiene knowledge		COR (95% CI)	AOR (95% CI)	p-value
	Good	Poor			
Gender					
Male	45	104	0.39 (0.23–0.68)*	0.38 (0.21–0.71)**	**0.002**
Female	47	43	1	1	
Age					
≤ 27 years	66	88	1	1	
> 27 years	26	59	0.59 (0.33–1.03)*	0.60 (0.31–1.19)	0.144
Marital status					
Not married	42	72	1.14 (0.68–1.93)		
Married	50	75	1		
Monthly income					
≤ 2000 ETB	35	71	1		
> 2000 ETB	57	76	1.52 (0.89–2.59)		
Educational status					
Primary	49	114	1	1	
Secondary and above	43	33	3.03 (1.72–5.32)*	2.26 (1.18–4.33)**	**0.014**
Job type					
Butcher	33	54	1	1	
Helper	22	45	1.26 (0.69–2.32)	0.70 (0.30–1.64)	0.411
Cooker	37	48	0.80 (0.43–1.46)*	0.49 (0.24–1.08)	0.078
Supervision by health professionals					
Yes	50	53	2.11 (1.24–3.59)*	3.05 (1.53–6.07)**	**0.002**
No	42	94	1	1	
Work experience					
**< **5 years	40	80	1	1	
5–10 years	41	55	1.83 (0.74–4.52)*	1.57 (0.50–4.75)	0.453
> 10 years	11	12	1.23 (0.49–3.06)	0.73 (0.21–2.51)	0.620
Meat hygiene training					
Yes	43	48	1.81 (1.06–3.09)*	1.97 (1.04–3.75)**	**0.038**
No	49	99	1	1	
Working hour per day					
≤ 8 hours	11	14	1.29 (0.56–2.98)		
> 8 hours	81	133	1		

Significant values are in bold.

*AOR:* Adjusted odds ratio, *CI:* confidence interval, *COR:* Crude odds ratio, *p-value < 0.25 in the bivariable analysis, **p-value < 0.05 in the multivariable analysis.

### Factors that associated with meat hygiene practice among meat handlers

In bivariable logistic regression analysis, variables such as gender, educational status, work experiences, supervision by health workers, meat hygiene knowledge level, regular medical check-up, and meat hygiene training were significantly associated with meat hygiene practices, with a p-value less than 0.25. Finally, in multivariable logistic regression, the independent variables that were significantly associated with meat hygiene practices at a p-value less than 0.05 were educational level, work experiences, supervision by health workers, and meat hygiene training.

Accordingly, those with 5–10 years of work experience are 4.31 times more likely to practice meat hygiene practices than their counterparts [AOR 4.31, 95% CI: 1.29–14.34, p-value = 0.010], those with secondary and higher education are 2.58 times more likely to practice meat hygiene practice than those with primary education [AOR 2.58, 95% CI: 1.25–5.3, p-value = 0.017], those who were supervised by health professionals were 4.01 times more likely to practice meat hygiene than those who were not supervised by health professionals [AOR 4.01, 95% CI: 1.85–8.68, p-value = 0.000], and those who had received meat hygiene training were 2.20 times more likely to practice meat hygiene than those who had not been trained [AOR 2.20, 95% CI: 1.13–4.27, p-value = 0.020] (Table 6).

**Table 6:** Factors associated with the meat hygiene practice among meat handlers, in Gedeo zone, Southern Ethiopia, 2022 (n = 239).

Variables	Meat hygiene practice		COR (95% CI)	AOR (95% CI)	p-value
	Good	Poor			
Gender					
Male	33	116	0.66 (0.37–1.20)*	1.11 (0.55–2.22)	0.774
Female	27	63	1	1	
Age					
≤ 27 years	41	113	1		
> 27 years	19	66	1.26 (0.68–2.35)		
Income					
≤ 2000 ETB	27	79	1		
> 2000 ETB	33	100	1.04 (0.57–1.86)		
Educational status					
Primary	28	135	1	1	
Secondary and above	32	44	3.51 (1.90–6.45)*	2.58 (1.25–5.31)**	**0.010**
Marital status					
Unmarried	25	89	1		
Married	35	90	1.38 (0.77–2.50)		
Supervision by health professionals					
Yes	39	64	3.34 (1.81–6.16)*	4.01 (1.85–8.68)**	**0.000**
No	21	115	1	1	
Regular medical-check-up					
Yes	29	55	2.11 (1.16–3.83)*	1.10 (0.52–2.30)	0.808
No	31	124	1	1	
Work experience					
< 5 years	24	96	1	1	
5–10 years	25	71	3.67 (1.44–9.32)*	14.31 (1.29–14.34)**	**0.017**
> 10 years	11	12	2.60 (1.02–6.64)*	2.43 (0.73–8.05)	0.146
Working hour per day					
≤ 8 hours	6	19	1		
> 8 hours	54	160	0.94 (0.36–2.46)		
Meat hygiene training					
Yes	35	56	3.07 (1.68–5.62)*	2.20 (1.13–4.27)**	**0.020**
No	25	123	1	1	
Meat hygiene knowledge level					
Good	16	76	2.03 (1.06–3.87)*	1.18 (0.43–3.23)	0.745
Poor	44	103		1	
Attitude					
Good	22	51	1.45 (0.78–2.69		
Poor	38	128	1		

Significant values are in bold.

*AOR:* Adjusted odds ratio, *CI:* confidence interval, *COR:* Crude odds ratio, *p-value < 0.25 in the bivariable analysis, **p-value < 0.05 in the multivariable analysis.

## Discussion

The aim of this study was to determine food safety knowledge, practices and associated factors among meat handlers. According to the findings of this study, 38.5% [95% CI: 32.2–44.8%] and 25.1% [95% CI: 19.7–30.5%] of meat handlers have good food safety knowledge and practices, respectively. This study's finding is higher than studies conducted in Amhara National Regional State (11.1%)^19^, Bangladesh (20%)^16^, and Jigjiga Town, Ethiopia (22%)^21^ among meat handlers. However, a study in Malaysia found that 61.7% of abattoir employees had a good knowledge of food safety^9^, and a study in Lagos State, Nigeria found that 71.1% of meat handlers had a good knowledge^17^, both of which were higher than the result of this study.

This study finding is consistent with a study conducted in Amhara National Regional State, which found that 25.7% of butcher shop and abattoir workers practiced good food safety practices^19^, as well as a study conducted in slaughter houses in north-central Nigeria (25.5%)^18^. However, the finding of this study is higher than those of a studies conducted in Jigjiga Town, Ethiopia (1.1%)^21^, Bangladesh (16.3%)^16^, and in Bishoftu City, Ethiopia (16.3%)^20^. On the contrary, this study finding is lower than those of studies conducted among butcher shops in Gondar town (66.4%)^14^, meat handlers in North Shewa Zone, Oromia, Ethiopia (51.3%)^27^, among meat handlers in Lagos State, Nigeria (66.6%)^17^, and abattoir workers in Malaysia (77.7%)^9^. The discrepancy in food safety practice level could be attributed to differences in the study tool employed, the period of the study, and variations in the study population's socio-demographic and economic status.

Gender, educational status, health worker supervision, and food safety training were found to be significantly correlated with meat handlers' meat hygiene knowledge. In this study, the male gender was found to be significantly (p = 0.002) associated with meat hygiene knowledge. Male meat handlers were 61% less likely than females to have meat hygiene knowledge. This study finding is supported by a studies conducted in Saudi Arabia^31^ and Bangladesh^32^. However, this study finding is contradicted by studies conducted among meat handlers in Bishoftu City, Ethiopia^20^ and Bangladeshi^33^, which found that male food handlers have a higher level of meat hygiene knowledge than females. Variations in results could be explained by differences in the study population, sample size, study settings, and socio-cultural status of study participants.

According to the findings of this study, secondary or higher education is a predictor of maximizing meat hygiene knowledge. Meat handlers with a secondary education or higher were 2.26 times more likely than their counterparts to have a good level of meat hygiene knowledge in this study. This study finding is in agreement with those of studies conducted in Chitwan, Nepal^29^, in Amathole District, Eastern Cape Province, South Africa^34^, and in Bangladesh^16^. The possible explanation is that education increased workers' awareness of food safety practices in the workplace while also increasing their exposure to mass media and other information sources.

According to the findings of this study, the majority of meat handlers (61.9%) had not received meat hygiene training. This study's findings are comparable to those of studies conducted in two Bangladeshi districts (85%)^35^, in Gondar Town, Ethiopia (66.04%)^36^, and in Mekelle City, Ethiopia (61.5%)^37^.

According to the literature, food handler training in food hygiene is important for providing safe food to consumers^38^, and handling food without food hygiene training increases the risk of cross contamination^29^. According to the current study, meat handlers who received meat hygiene practices training were 1.97 times more likely to have higher meat hygiene knowledge than those untrained ones. This study finding is supported by study conducted in Bangladesh^16^, in Chitwan, Nepal^29^. Study finding from abroad reported that training can improve knowledge of employee^39^ that is why in this study meat handlers who received meat hygiene training have higher meat hygiene knowledge. Furthermore, a study conducted in Malaysia revealed that food hygiene training improves food handlers' food safety knowledge and practice^40^. This could be due to food handlers who did receive food hygiene training having the necessary knowledge and experience as a result of professional advice they received from trainees during training. Furthermore, training may have an effect on changing the behaviors of food handlers to adhere to food safety practices.

In this study, regular supervision by a health professional was found to be significantly associated with meat hygiene knowledge (p = 0.002). Meat handlers who were supervised by health workers were 3.05 times more likely to be familiar with meat hygiene practices than those who were not. This study's findings are supported by a study conducted among food service workers in Bangladeshi hospitals^33^. The possible explanation is that during supervision, health workers and managers may provide on-the-job training on food safety practices, which is why workers supervised by health workers or managers have good knowledge of meat hygiene practices.

In this study educational status, supervision by health care workers, work experiences and meat hygiene training were factors significantly associated with meat hygiene practice among meat handlers. Meat handlers with educational status of secondary and above were 2.58 times more likely practice meat hygiene practices than the counterparts. This study finding is supported with study conducted in North Shewa Zone, Oromia, Ethiopia^27^, study conducted in Kenya^41^, study conducted among meat-handlers in Metropolitan City of Kathmandu, Nepal^11^. The probable explanation is that those with a higher level of education can understand some of the regulations and instructions pertaining to meat hygiene practices.

In this study, supervision by a health professional was found to be significantly associated with meat hygiene practice (p = 0.000). Meat handlers who were supervision by health professionals were 4.01 times more likely to be familiar with meat hygiene practices than those who were not. This study finding is inline by studies conducted among food handlers in Arba Minch^42^, in Gondar city^43^ and among meat handlers in Lagos State, Nigeria^17^. The possible explanation might by during supervision by health works give awareness creation training for meat handlers by giving practical support and feedback for meat handlers.

Meat handlers with 5–10 years of experience were 14.31 times more likely than workers with less than 5 years of experience to practice meat hygiene practices. This study finding is supported by studies conducted in Lagos State, Nigeria^17^, in north-central Nigeria^44^, in North Shewa Zone, Oromia, Ethiopia^27^ and study conducted in Food Handlers of Fiche Town, North Shewa Zone, Ethiopia^45^ which found that experienced workers had good practice towards meat hygiene practices. The possible explanation could be that experienced food handlers may have better knowledge and skills regarding meat hygiene practice.

In this study, 38.1% of meat handlers were trained in meat hygiene practices, which was higher than studies conducted in North Shewa Zone, Oromia, Ethiopia among Meat Handlers (12.1%)^27^, in Nepal among meat handlers (7%)^11^, in Dharan Municipality of Eastern Nepal (30.4%)^46^, in the Dhaka megacity of Bangladesh among red meat handlers (21.25%)^47^.

In this study, meat handlers who received meat hygiene training were 2.20 times more likely to practice meat hygiene than those who did not receive meat hygiene training. This study's findings are supported by studies conducted in the Gamo Gofa Zone of Southern Ethiopia^42^, Bishoftu City, Ethiopia among meat handlers^20^, in Kenya^41^, in north-central Nigeria^44^, in Gondar city^43^, in Bangladesh among meat handlers^16^, and in eastern Cape Province, South Africa among slaughter house workers^34^. A possible explanation is that meat handlers become aware of proper meat hygiene practices as a result of meat hygiene training.

These findings suggest that good meat hygiene knowledge and practices could serve as an important component in promoting proper meat handling practices. As a result, more emphasis should be given to educating meat handlers, providing training to meat handlers working in butcher shops, and conducting frequent supportive supervision by health professionals, as well as considering these aspects and developing policy and regulations to guide and monitor butcher shops across the country in order to protect communities from potential meat-borne disease.

## Conclusion and recommendation

In this study, less than half of the study participants have good meat hygiene knowledge and practices. Educational level, gender, having meat hygiene training, and having supportive supervision by health professionals were factors significantly associated with good meat hygiene knowledge among meat handlers, whereas work experience, educational level, having supportive supervision by health professionals, and having meat hygiene training were factors significantly associated with good meat handling practice among meat handlers. To improve meat hygiene practices, meat handlers should follow the guidance and recommendations of health professionals and regulatory bodies, such as having regular medical checkups, keeping proper personal hygiene, and enhancing their knowledge and practice of meat hygiene. Butcher shop owners should provide sanitation and hygiene facilities, as well as train meat handlers and hire meat handlers with a higher level of education, to maintain appropriate meat hygiene practices among meat handlers and the cleanliness of butcher shops. Healthcare professionals and regulatory bodies should conduct frequent sanitary inspections or supportive supervisions and provide immediate feedback to butcher shops, as well as organize meat handler trainings and regular medical checkups in collaboration with health facilities and other stakeholders. To prevent meat-borne disease, the Zonal health department or Woreda Health Office should regulate, supervise, and coordinate the implementation of different hygienic meat handling standards, guidelines, and policies. Furthermore, laboratory-based and follow-up studies with a large sample size should be conducted to show a cause-effect relationship in order to prevent meat-borne infections.

## Data Availability

The datasets generated during and/or analyzed during the current study are available from the corresponding author on reasonable request.

## Abbreviations

AOR: Adjusted odds ratio
CI: Confidence interval
COR: Crude odds ratio
DALYs: Disability-adjusted life-years
IRB: Institutional Review Board
SD: Standard deviation
